# Stand-Alone Oblique Lumbar Interbody Fusion (OLIF) for the Treatment of Adjacent Segment Disease (ASD) after Previous Posterior Lumbar Fusion: Clinical and Radiological Outcomes and Comparison with Posterior Revision Surgery

**DOI:** 10.3390/jcm12082985

**Published:** 2023-04-20

**Authors:** Massimo Miscusi, Sokol Trungu, Luca Ricciardi, Stefano Forcato, Amedeo Piazza, Alessandro Ramieri, Antonino Raco

**Affiliations:** 1 NESMOS Department, Sant’Andrea Hospital, Sapienza University of Rome, 00185 Rome, Italy; 2 Neurosurgery Unit, Cardinale G. Panico Hospital, 73039 Tricase, Italy; 3 Department of Orthopedics, Faculty of Pharmacy and Medicine, Sapienza University of Rome, 00185 Rome, Italy

**Keywords:** oblique lumbar interbody fusion (OLIF), adjacent segment disease (ASD), revision lumbar surgery, failed back surgery, minimally invasive spine surgery

## Abstract

**Background**: Radiological evidence of adjacent segment disease (ASD) has been reported to have a prevalence of more than 30% and several risk factors have been reported. The aim of this study is to evaluate the clinical and radiological outcomes of patients with symptomatic ASD treated with stand-alone OLIF and compare results with a posterior revision surgery cohort. **Methods**: This is a retrospective case-control study. Clinical-patient-reported outcomes were obtained at preoperative, postoperative and final follow-up visits using the Short Form (SF-36) scale, the Oswestry Disability Index (ODI) and the visual analog scale (VAS). Radiological measures include lumbar lordosis (LL), segmental lordosis (SL), pelvic incidence-lumbar lordosis (PI-LL) mismatch, segmental coronal Cobb angle and intervertebral disc height (DH). The data are compared with a retrospective series of patients that underwent a posterior revision surgery for ASD. **Results**: Twenty-eight patients in the OLIF group and 25 patients in the posterior group meet inclusion criteria. The mean ages at the time of the surgery are 65.1 years and 67.5, respectively. The mean follow-up time is 36.1 months (range of 14–56). The clinical outcomes significantly improve from preoperative values from the surgery in both groups. The radiological parameters are significantly improved postoperatively and were maintained at the last follow-up in both groups. A statistically significant difference is observed between the two groups for minor complication rate, length of surgery, blood loss and DH restoration. **Conclusions**: Stand-alone OLIF is an effective and safe technique with low morbidity and complication rates for the treatment of selected patients with symptomatic ASD following a previous lumbar fusion.

## 1. Introduction

The number of lumbar fusions has increased worldwide in the last decades as the standard surgical treatment for a variety of spine conditions, ranging from degenerative disc disease (DDD) to high-grade spondylolisthesis, and different approaches for achieving successful fusion have been suggested [1,2,3,4,5]. Consequently, the incidence of adjacent segment disease (ASD), a potential long-term complication of spinal fusion characterized by the degeneration of a mobile segment adjacent to the last instrumented vertebra, is increasing significantly.

Several studies report that the radiological evidence of ASD has a prevalence of more than 30%, and several risk factors, both patient or surgery-related, for its development have been reported [6,7,8,9,10]. The incidence of symptomatic ASD requiring reoperation ranges from 1.7 to 9%, increasing substantially during years after surgery [11]. Although there is no established standard treatment for ASD, it is typically surgically treated with posterior decompression and extension of instrumented levels [12,13].

However, revision surgery is related to higher morbidity and is more technically challenging due to scar tissue from the previous surgery and prior instrumentation [14,15,16,17]. Other possible alternatives to avoid complications are the anterior/lateral approaches, a minimally invasive techniques for treating ASD that preserves posterior ligamentous structures, achieving indirect decompression and a solid interbody fusion of the unstable segment, minimizing morbidities compared to traditional posterior approach [18,19,20,21,22,23,24,25]. 

The aim of this study is to evaluate the clinical and radiological outcomes of patients with symptomatic ASD treated with stand-alone OLIF and to compare the results with a retrospective series of patients that underwent a posterior revision surgery.

## 2. Materials and Methods

### 2.1. Study Design and Guidelines

This is a retrospective control study from a single institution. According to the study design and the non-modification of the standard of care, the IRB or ethical committee approval was not required. All of the patients expressed written consent to the surgical procedure after receiving appropriate information. The data reported have been completely anonymized. Therefore, this study is perfectly consistent, in all of its aspects, with WMA Helsinki Declaration of Human Rights.

### 2.2. Patient Population

This is a retrospective review of patients who presented with symptomatic ASD in the lumbar spine and who underwent stand-alone OLIF (OLIF group) or posterior revision surgery (posterior group) between December 2015 and December 2019 in our institutions.

The inclusion criteria for both groups are as follows: symptomatic ASD in a patient with previous posterior lumbar fusion (consequent low back pain, lower extremity radiculopathy, neurogenic claudication) demonstrated by radiological images that show an adjacent level pathology (DDD, spondylolisthesis, segmental kyphosis, foraminal stenosis); and unresponsiveness to conservative therapy for over 3 months; follow-up >12 months. The exclusion criteria for the OLIF group are as follows: ≥Grade 2 spondylolisthesis (Meyerding classification) [26]; severe stenosis grade C and D (defined by a neuroradiologist based on a classification on an MRI as described by Schizas et al.) [27]; unfavorable anterior vascular anatomy; recent trauma; history of retroperitoneal surgery in the previous year; and <12 months follow-up.

### 2.3. Surgical Technique

#### 2.3.1. OLIF Group

The patient is put in the right lateral decubitus position and a 4 cm skin incision, centered on the spinal segment to expose, is made in the left lateral abdominal region; the incision is parallel to the fibers of the external oblique muscle, 5 cm in front of the projection of the disk anterior wall. Using blunt scissors and finger dissection, external oblique, internal oblique and transverse abdominal muscles are then progressively dissected, and the retroperitoneal space is accessed by the blunt mobilization of the peritoneal content anteriorly.

The psoas muscle is identified and the first blunt retractor is posed just in front of its tendon. The sympathetic chain and the ureter are anteriorly mobilized, together with the peritoneum content. Another two or three blunt retractors are used to expose the disk space and are fixed with pins to the above and below vertebral body. The discectomy is made, and after insertion of a trial, the final cage, packed with bone chips or bone substitute, is positioned under A-P and L-L fluoroscopy. Segmental vessels usually do not need to be ligated unless the vertebral body needs to be exposed. If the disc space is obstructed by veins, these must be dissected and mobilized or ligated. A drainage is left in the retroperitoneal space and the abdominal muscles and superficial planes are closed sequentially. Titanium porous 3D interbody cages with a footprint of 30 × 29 mm and different range of heights (8–14 mm) and lordosis (0–14°) (Ortigia; Tsunami Medical srl, Modena, Italy) are used in this cohort. For intervertebral fusion, we used a different type of bone substitutes (bovine mineralized bone plus collagen; calcium phosphate granules or paste; and paste of demineralized bone matrix). No lumbar orthosis was used after the surgery.

#### 2.3.2. Posterior Group

The patient is positioned in a prone position. A midline incision is made with the exposure of previous instrumentation and the levels to be instrumented. A standard open technique for screw placement, laminectomy and bilateral posterior lumbar interbody fusion (PLIF) was performed in all of the patients of this group. Different implants were used based on the type of instrumentation used in the previous surgery.

### 2.4. Clinical and Radiological Outcomes

General and neurological conditions, as well as the quality of life, were evaluated at admittance (preoperative parameters), 6 weeks after surgery and final follow-up, using self-reported measures as the visual analog scale (VAS), the Oswestry Disability Index (ODI) and the short-form SF-36 scores. The preoperative radiological evaluation includes lumbar standing and dynamic X-rays to evaluate spino-pelvic parameters and spinal stability, as well as an MRI and CT to detect the course of great vessels. The radiological evaluation on the standing X-rays was based on modifications of the lumbar lordosis (LL), segmental lordosis (SL), pelvic incidence and lumbar lordosis mismatch (PI-LL mismatch), disc height (DH) and segmental coronal Cobb angle (cCobb), compared with the baseline. The interbody fusion was evaluated on postoperative X-rays and CT scan (presence of bridging bone through or around the implants connecting the adjacent vertebral bodies, less than 3 mm of translation on dynamic X-rays). Perioperative complications were classified as major and minor as described by Glassman et al. [28]. Any new postoperative motor and/or sensory deficits identified on examination were evaluated as minor or major complications depending on if they resolved within 3 months of surgery or not, respectively.

### 2.5. Statistical Analysis

The statistical analysis was performed using SPSS v.18. Paired *t* tests were used to compare outcomes between preoperative and postoperative measurements. The Fisher exact test, chi-squared test or 2-sample z-test was used to compare the variables between groups. The level of significance was set at *p* < 0.05.

## 3. Results

### 3.1. Demographical and Surgical Data

A total of 28 patients underwent stand-alone OLIF for ASD during the study period and met the inclusion criteria for our analysis. There are 11 (39.3%) women and 17 (60.7%) men. The average age at the time of surgery is 65.1 ± 6.8 years (range 54–75). The average follow-up is 36.1 ± 14 months (range 14–56). All patients presented with back pain, radiculopathy (39.3%) and neurogenic claudication (28.6%). The most common co-morbidity is cardiovascular diseases (67.9%), followed by diabetes mellitus (42.8%), obesity (32.1%) and respiratory diseases (28.6%). Twelve patients (42.9%) are smokers. Two patients presented with ASA Class I (7.1%), whereas 11(39.3%), 14 (50%) and 1 (3.6%) patients presented with ASA Classes II, III, and IV, respectively. The most common pathological level was L3–L4 (53.4%), followed by L2–L3 (43.3%) and L1–L2 (3.3%). Twenty-six patients (92.9%) underwent a single-level OLIF and 2 patients (7.1%) underwent two-levels OLIF. The most common radiological presentation of ASD was DDD (78.6%), followed by segmental kyphosis (53.6%), spondylolisthesis (42.9%) and foraminal stenosis (35.7%). The mean length of surgery is 67 ± 15.6 min (range 55–130) with an average of 55 ± 13.9 mL (range 40–100 mL) of estimated blood loss (EBL). The mean length of stay (LOS) is 2 days (range 1–4) with a mean time of 24 h of postoperative mobilization. No complications were noted intraoperatively. All patients were discharged home. A total of 25 patients underwent posterior revision surgery during the same period with similar demographic characteristics. However, the mean length of surgery, ELB, LOS and postoperative mobilization are 192.8 min ± 55.6, 308.8 mL ± 108.7, 4 days (2–7) and 2 days (1–5), respectively. All of the patients and operative characteristics from both groups are summarized in Table 1 and Table 2.

### 3.2. Clinical and Radiological Outcomes

All of the clinical outcomes improved significantly after surgery and remained stable during the follow-up in both groups. ODI scores improved from 53.9 to 22.8 at follow-up (*p* < 0.05). The SF-36 scores (preop 36.9 vs. 70.7 at follow-up, *p* < 0.05) and VAS scores (preop 7.9 vs. 2.5 at follow up, *p* < 0.05) improved significantly after surgery. Similarly, in the posterior group the means of all of the clinical outcomes improved significantly after surgery. All of the patients’ clinical outcomes are summarized in Table 3.

**Figure 1:**
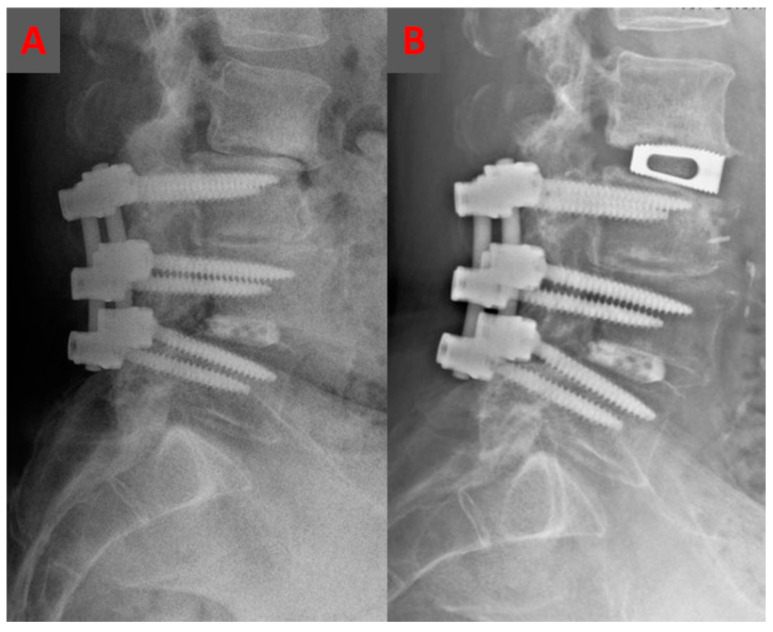
An illustrative case showing a preoperative lateral lumbar radiograph of a 69-year-old man following a posterior L3–L5 fusion 4 years ago and a severe disc degeneration, and retrolisthesis present at L2–L3 (**A**). The patient underwent a stand-alone oblique lumbar interbody fusion at L2–L3 with restoration of lumbar lordosis and intervertebral disc heights (**B**).

Lumbar (preop −39.4 vs. −46.1 at follow-up, *p* < 0.05) and segmental lordosis (preop −5.4 vs. −9.9 at follow-up, *p* < 0.05) improved significantly after surgery and were maintained at the follow-up. Similarly, the PI-LL mismatch (preop 15.2 vs. 10.3 at follow-up, *p* < 0.05) and the disc height (preop 5.3 mm vs. 8.4 mm at follow-up, *p* < 0.05) improved significantly after surgery. The segmental coronal angle improved after surgery but not significantly (preop 4.8 vs. 4.1 at follow-up, *p* = 0.155). Equally, in the posterior group, all of the radiological outcomes improved after surgery. The fusion rate was 92.9% in OLIF group and 92% in the posterior group. All of the patients’ radiological outcomes are summarized in Table 4. An illustrative case is presented in Figure 1.

### 3.3. Complications and Reoperation Rate

**Figure 2:**
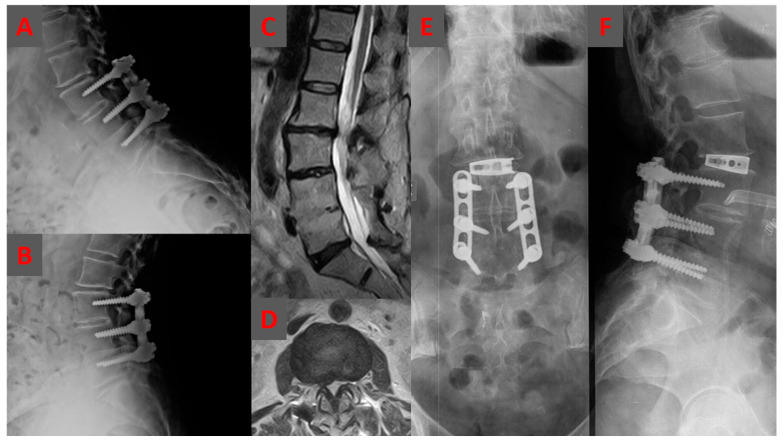
An illustrative case showing a preoperative dynamic lateral lumbar radiograph of a 72-year-old woman following a posterior L3–L5 fusion and a disc degeneration, and retrolisthesis present at L2–L3 (**A**,**B**). A T2-weighted sagittal (**C**) and axial (**D**) MRI scan confirming the disc degeneration and showing a right foraminal stenosis. Anterior (**E**) and lateral (**F**) radiograph showing the stand-alone oblique lumbar interbody fusion at L2–L3 with cage subsidence.

In the OLIF group, no major complications were noted post-operatively or during the follow-up. One minor complication was observed; one patient experienced a left L3 paresthesia with complete resolution within 2 months. Two patients required reoperation (7.1%). The first patient was a 64 years old woman who underwent an L3–L4 OLIF after a previous L4–S1 open laminectomy and postero-lateral fusion. During the follow-up, her symptoms (neurogenic claudication) did not improve and the patients had a revision surgery after 6 months with a laminectomy and an extension of posterior stabilization. The second patient, a 73 years old man who underwent an L2–L3 OLIF, developed back pain 6 weeks after surgery. The X-rays and a CT scan showed a cage subsidence and he underwent an L2–L3 laminectomy and an extension of stabilization. Yet, one patient who underwent L2–L3 OLIF after a previous L3–L5 posterior fusion had an asymptomatic radiological cage subsidence that did not need a revision surgery (Figure 2, below).

In the posterior group, one major complication (4%) was observed; one patient experienced a permanent left L3 nerve root motor deficit. Five minor complications (20%) were observed: 2 patients with a dural tear and 3 patients with a superficial wound infection. One patient (4%) needed a revision surgery to repair a CSF leakage.

### 3.4. Comparision with Posterior Group

No significant differences are found between the two groups in terms of patient characteristics, clinical and radiological outcomes. However, statistically significant differences can be observed between the two groups related to operative characteristics, postoperative VAS score and minor complication rate. Particularly, there is a significant difference in the mean length of the surgery (67.1 ± 15.7 vs. 192.8 ± 55.6, *p* < 0.0001), the mean ELB (55.2 ± 13.9 vs. 308.8 ± 108.7, *p* < 0.0001), the mean LOS (2 vs. 4 days, *p* < 0.0001) and the mean time of postoperative mobilization (1 vs. 2 days, *p* < 0.0001) in the OLIF group. Furthermore, the 6-weeks postoperative VAS score (3.3 ± 1.0 vs. 3.9 ± 1.1, *p* = 0.04), the minor complication rate (3.6% vs. 20%, *p* = 0.03) and the DH (8.4 ± 1.1 vs. 7.8 ± 0.9, *p* = 0.04) are significantly better in the OLIF group.

## 4. Discussion

Nowadays, the prevalence of lumbar fusion procedures has progressively increased and consequently ASD has become a frequent pathology. Radiological ASD, symptomatic or not, has a wide reported incidence ranging from 5% to 92%, due to a lack of consensus in the radiological criteria used for its diagnosis [29,30,31]. The rate at which patients presenting with ASD require a second surgery is approximately 4% per year across various diagnoses, with a reported incidence of 16.5% at 5 years and 36.1% at 10 years [32].

Clinically, ASD presents as a new onset of substantial mechanical back and leg pain and sagittal or coronal imbalance, with a significative reduction of quality of life (QOL), and it represents an indication for the surgical revision of the primary fusion. The main goals of the surgical treatment of ASD are the arthrodesis of mobile segments, restoring the sagittal and coronal profile and the decompression of neural structures.

A more typical approach is to perform revision posterior surgery with both a laminectomy and the extension of the instrumentation and fusion to the rostral level(s). However, this method requires extensive soft tissue dissection to expose the previously implanted hardware, adding to the surgical blood loss and postoperative pain and prolonging recovery with high associated health care costs [33].

For example, Smorgick et al. find that the revision posterior surgery involves a mean blood loss of 1606 mL, 16% greater than in similar primary surgeries [16]. The surgeon will typically have to remove or extent the previous hardware construct, expanding the surgical enterprises. In addition, exposing the previous laminectomy site poses a higher risk of dural tears and CSF leakage due to postoperative scar tissue [34].

For these reasons, anterior/lateral approaches could be an effective alternative to avoid these complications. Minimally invasive surgical techniques to fuse and decompress the spinal column have become increasingly popular over the past decade [35,36,37].

Lateral lumbar interbody fusion (LLIF) is a technique which has been proposed for the treatment of ASD. It is a minimally invasive approach which requires a psoas muscle violation to reach the lateral aspect of the disk and vertebral bodies; through it, a large footprint cage can be inserted to obtain a solid single or multilevel inter-somatic fusion with a good segmental realignment [21,38]. The LLIF limits can be avoided by the use of a MIS ante-psoas approach, which could allow the same ability to insert a large footprint cage, but avoiding a lumbar plexus injury and allowing an easier dissection of the ALL. In this sense, OLIF represents a valid alternative MIS anterior approach for the treatment of ASD.

The OLIF approach was first described by Michael Mayer in 1997 and involves an MIS access to the disc space via a corridor between the peritoneum and psoas muscle [39]. Similarly to an LLIF approach, OLIF does not require posterior surgery, laminectomy, arthrectomy or the stripping of spinal or paraspinal musculature. It can be indicated in the case of pure DDD [40], treatment of complex lumbar degenerative deformities and for the treatment of segmental instability due to ASD [41,42,43,44,45].

This is currently the largest comparative study in the literature and it suggests that stand-alone OLIF is an effective option for symptomatic ASD with statistically significant postoperative improvements in clinical and radiographic outcomes. Moreover, our series of patients had a low reoperation rate without major complications. Comparing the two groups, OLIF is superior to posterior revision surgery in terms of the length of the surgery (*p* < 0.0001), ELB (*p* < 0.0001), the LOS (*p* < 0.0001) and the time of postoperative mobilization (1 vs. 2 days, *p* < 0.0001) in the OLIF group. Furthermore, the 6-weeks postoperative VAS score (*p* = 0.04), the minor complication rate (*p* = 0.03) and the DH (*p* = 0.04) are significantly better in the OLIF group.

These results compare favorably with those reported for revision posterior instrumented fusion [14,17], and for the use of the LLIF approach in ASD [20,34]. Only a few studies have evaluated the use of stand-alone OLIF as a treatment of symptomatic ASD. Jin et al. retrospectively assesses 12 patients who underwent a single level OLIF for ASD compared with 14 patients that underwent a posterior surgery [24]. The authors report a clinical improvement and that compared with posterior reoperation, OLIF results in a shorter operative time and hospital stay, less blood loss, and a lower risk of a dural injury. Zhu et al. report the same results in their study [23]. In our study, only one patient (4.3%) required a posterior decompression and instrumentation due to the persistence of symptoms, suggesting that in some cases, indirect decompression is not enough for clinical improvement.

The use of an adjunctive anterior plating seems not detrimental in enforcing the strength of primary stability [46,47].

Interestingly, a recent biomechanical study suggests that stand-alone anterior cages can be considered for an alternative treatment of ASD, but in case of severe instability, the circumferential approach seems to provide the most biomechanically stable construct [48]. In addition, several biomechanical studies suggest that the addition of a posterior instrumentation improves the stability and rigidity of the construction and reduces the risk of complications [49,50,51,52].

We use a large footprint 3D titanium lordotic cages without plating, and we obtain a good primary stability with a high fusion rate (92.9%) in all cases, as demonstrated by the postoperative X-rays. Usually, we prefer a middle-third endplate cage positioning that, in our opinion, shows a better restoration in DH and decreases the incidence of cage subsidence. Furthermore, compared with the posterior group, the DH restoration is better with OLIF cages. In only two of 28 cases (7.1%), we recurred to a second posterior surgery to obtain a good decompression and to enforce the stability after the partial demolition of posterior facets. These results seem to confirm that in the majority of cases, a pure anterior approach can be proposed.

A lordotic cage should always be used, but our experience suggests that the oversizing of cages, which is a common mistake in such an approach, generally leads to their subsidence (2 cases in our series), and the failure of the planned surgical strategy.

In term of feasibility, an OLIF approach can be proposed only to selected patients. An analysis of great vessels’ anatomy must be made, but experience suggests paying particular attention to the left iliac vein, which represents the only real enemy of such an approach. When the vein lies just in front of the target disk, the procedure should be avoided and LLIF is preferred.

### Limitations of the Study

There are some limitations to this study. First, the study was retrospectively conducted by case selection and is not randomized. Second, the follow-up period is short and the sample of patient is relatively small. Third, in case of severe stenosis, this approach may not provide an adequate decompression and a combined anterior/posterior approach is required in some cases. Nevertheless, future prospective randomized studies involving a long-term follow-up with a larger number of patients are required to clarify the advantages of OLIF over a posterior revision surgery for ASD treatment.

## 5. Conclusions

This is the largest comparative study in the current literature that evaluates the clinical and radiological outcomes following a stand-alone OLIF for patients with symptomatic ASD following a previous lumbar fusion, suggesting that it is a safe and effective treatment option with low complication and reoperation rates. Furthermore, compared with the posterior group, OLIF results in a shorter length of surgery, hospital stay and postoperative mobilization, less blood loss, better disc height restoration and a lower complication rate.

## Figures and Tables

**Table 1:** Patient characteristics.

	OLIF Group	Posterior Group
**Total N° of Patients**	28	25
Mean age ± SD, yrs (range)	65.1 ± 6.8 (54–75)	67.5 ± 5.9 (59–77)
Mean Follow-up ± SD, mos (range)	36.1 ± 14 (14–56)	32.6 ± 12.1 (14–56)
**Sex**		
Female	11 (39.3%)	11 (44%)
Male	17 (60.7%)	14 (56%)
**ASA Classification**		
I	2 (7.1%)	0
II	11 (39.3%)	11 (44%)
III	14 (50.0%)	12 (48%)
IV	1 (3.6%)	2 (8%)
V	0	0
**Smoking status**		
Smoker	12 (42.9%)	10 (40%)
Non-Smoker	16 (57.1%)	15 (60%)
Clinical presentation *		
Low back pain	28 (100%)	25 (100%)
Radiculopathy	11 (39.3%)	13 (60%)
Neurogenic claudication	8 (28.6%)	16 (64%)
Lower extremity weakness	0	4 (16%)
Comorbidity *		
Cardiovascular diseases	19 (67.9%)	16 (64%)
Diabetes Mellitus	12 (42.8%)	9 (36%)
Obesity	9 (32.1%)	8 (28%)
Respiratory diseases	8 (28.6%)	6 (24%)

* Patients may have multiple clinical presentation and comorbidities.

**Table 2:** Operative characteristics.

	OLIF Group	Posterior Group	*p* Value *
**Level of ASD**			
L1–L2	1 (3.3%)	2 (8%)	
L2–L3	13 (43.3%)	10 (40%)	
L3–L4	16 (53.4%)	13 (52%)	
**Levels treated**			
One level	26 (92.9%)	21 (84%)	
Two levels	2 (7.1%)	4 (16%)	
**ASD radiological presentation**			
DDD	22 (78.6%)	20 (80%)	
Segmental kyphosis	15 (53.6%)	12 (48%)	
Spondylolisthesis	12 (42.9%)	10 (40%)	
Foraminal stenosis	10 (35.7%)	8 (32%)	
**Mean length of surgery, min** **(range)**	67.1 ± 15.7 (55–130)	192.8 ± 55.6 (62–150)	**<0.0001**
**Mean length of stay (LOS), days (range)**	2 (2–4)	4 (2–7)	**<0.0001**
**Mean time of postoperative mobilization, days (range)**	1 (1–3)	2 (1–5)	**<0.0001**
**Estimated blood loss (ELB), mL** **(range)**	55.2 ± 13.9 (40–100)	308.8 ± 108.7 (180–500)	**<0.0001**
**Complication Rate**			
Major	0	1 (4%)	0.14
Minor	1 (3.6%)	5 (20%)	**0.03**
**Reoperation Rate**	2 (7.1%)	1 (4%)	0.31

ASD, adjacent segment degeneration; DDD, degenerative disc disease; patients may present with multiple ASD features; * *p* value between two groups. In bold font statistically significant results.

**Table 3:** Clinical outcomes.

	Mean ± SD		
	OLIF Group	Posterior Group	*p* Value *
**Visual Analogue Scale (VAS)**			
Preoperative	8.2 ± 1.3	7.9 ± 1.2	0.39
Postoperative (6 weeks)	3.3 ± 1.0	3.9 ± 1.1	**0.04**
Follow-up at 12 months	2.6 ± 0.8	2.8 ± 0.9	0.40
*p* value (pre vs. follow-up)	**<0.05**	**<0.05**	
**Oswestry Disability Index (ODI)**			
Preoperative	53.6 ± 15.2	55.1 ± 14.1	0.71
Postoperative (6 weeks)	27.5 ± 7.2	28.9 ± 6.8	0.47
Follow-up at 12 months	22.7 ± 6.5	23.2 ± 5.9	0.77
*p* value (pre vs. follow-up)	**<0.05**	**<0.05**	
**SF-36 (Physical and Mental)**			
Preoperative	36.5 ± 6.2	38.1 ± 6.0	0.35
Postoperative (6 weeks)	66.2 ± 6.3	68.3 ± 5.9	0.22
Follow-up at 12 months	70.5 ± 5.4	69.6 ± 6.1	0.57
*p* value (pre vs. follow-up)	**<0.05**	**<0.05**	

* *p* value between two groups. In bold font statistically significant results.

**Table 4:** Radiological outcomes.

	Mean Value ± SD		
	OLIF Group	Posterior Group	*p* Value *
**Lumbar lordosis (LL)**			
Preoperative	−39.4 ± 7.8	−37.2 ± 6.8	
Postoperative (6 weeks)	−47.2 ± 8.9	−46.2 ± 7.9	
Follow-up at 12 months	−46.1 ± 9.1	−45.6 ± 8.7	0.84
*p* value (pre vs. follow-up)	**<0.05**	**<0.05**	
**Segmental lordosis (SL)**			
Preoperative	−5.4 ± 5.7	−6.6 ± 5.9	
Postoperative (6 weeks)	−11.4 ± 4.7	−11.8 ± 3.9	
Follow-up at 12 months	−9.9 ± 4.9	−10.4 ± 4.2	0.69
*p* value (pre vs. follow-up)	**<0.05**	**<0.05**	
**PI-LL mismatch**			
Preoperative	15.2 ± 6.9	13.9 ± 6.1	
Postoperative (6 weeks)	9.7 ± 4.6	9.9 ± 4.8	
Follow-up at 12 months	10.5 ± 5.1	11.1 ± 4.9	0.67
*p* value (pre vs. follow-up)	**<0.05**	**<0.05**	
**Segmental coronal angle**			
Preoperative	4.8 ± 1.8	5.4 ± 1.5	
Postoperative (6 weeks)	4.2 ± 1.7	4.9 ± 2.0	
Follow-up at 12 months	4.1 ± 2.0	4.6 ± 1.7	0.33
*p* value (pre vs. follow-up)	0.155	0.08	
**Disc height (DH)**			
Preoperative	5.3 ± 1.2	5.1 ± 1.0	
Postoperative (6 weeks)	8.7 ± 1.0	8.1 ± 1.2	
Follow-up at 12 months	8.4 ± 1.1	7.8 ± 0.9	**0.04**
*p* value (pre vs. follow-up)	**<0.05**	**<0.05**	
**Fusion Rate (n, %)**	**26 (92.9%)**	**23 (92%)**	0.90

* *p* value between two groups. In bold font statistically significant results.

## Data Availability

Not applicable.
